# Supplementary material for: How the public imagines the energy future: Exploring and clustering non-experts’ techno-economic expectations towards the future energy system
Source: PLoS One. 2020 Mar 5;15(3):e0227369. doi: 10.1371/journal.pone.0227369 (PMC7058329; doi:10.1371/journal.pone.0227369)
Supplement: S1 Appendix — The Appendix contains the following tables: S1 Table Socio-demographic sample description and comparison with Swiss population. S2 Table Overview of cluster solutions. S3 Table Internal consistency of energy system expectation scales (using Cronbach’s α). S4 Table Differences between the energy system expectation clusters with respect to socio-demographics, future orientation and political orientation. S5 Table Differences between the energy system expectation clusters with respect to energy attitudes and voting behaviour in the ES2050 referendum. S6 Table Questionnnaire with survey items (german and English Translation). (PDF) [file pone.0227369.s001.pdf]

S1_Appendix: Supporting information for the paper *How the public imagines the energy future: Exploring and clustering non-experts' techno-economic expectations towards the future energy system*

This file contains a sample description (including a comparison with the Swiss population), an overview of different cluster solutions, the internal consistency of energy system expectation scales (using Cronbach's α), the complete ANOVA tables as well as the questionnnaire (German original and English Translation).

1. Sample description and comparison with Swiss population

Table S1. Socio-demographic sample description and comparison with Swiss population

		**Survey sample** N=797	**Switzerland 2017** (Swiss Federal Office of Statistics)[1] 8.54mil.
**Demographics**	Age (mean)	44.0	42.4
	Female	50.0%	50.4%
	University degree	22.3%	27.0%
**Party preference**	Swiss People's party (SVP)	27.6%	29.4%
	Social Democratic Party (SP)	16.7%	18.8%
	Liberal Democratic party (FDP)	12.5%	16.4%
	Other parties	43.2%	35.4%

2. Overview of cluster solutions

Table S2. Overview of clustering results with different number of clusters.

3-Cluster solution	4-Cluster solution	5-Cluster solution
Cluster 1 (*N*=137)	Cluster 1 (*N*=137)	Cluster 1 (*N*=137)
Cluster 2 (*N*=200)	Cluster 2 (*N*=200)	Cluster 2 (*N*=200)
Cluster 3 (*N*=303)	Cluster 3 (*N*=122)	Cluster 3 (*N*=122)
	Cluster 4 (*N*=181)	Cluster 4 (*N*=93)
		Cluster 5 (*N*=88)

3. Report of confirmatory factor analyses

We based our choice of survey items on the literature on expectations and transition studies. In order to verify the internal consistency among included survey items, we computed Cronbach's α, a measure of inter-item reliability that ranges between 0-1. The Cronbach's α values our measures are well within the conventional range of 0.6-1, indicating that the included items are

[1] Source: https://www.bfs.admin.ch/bfs/de/home/statistiken/bevoelkerung/stand-entwicklung/bevoelkerung.html [Accessed: 15.08.2019]

reasonably clustered closely with each other as we hypothesized. Therefore, instead of empirically adjusting the included items post-hoc, we proceeded with the initial set of items as we hypothesized based on the literature. Both the TransitionExtent scale (consisting of three expectations) and the SystemState scale (consisting of seven expectations) returned only a single eigenvalue that is greater than 1, verifying that there is only one underlying factor beneath our choice of items as we hypothesized.

Table S3. Internal consistency of energy system expectation scales (using Cronbach's α)

**TransitionExtent**
Cronbach's α = 0.68<br>Number of eigenvalues > 1 = 1
**Items included:**
Renewables<br>Efficiency<br>Electric vehicles
**SystemState**
Cronbach's α = 0.65<br>Number of eigenvalues > 1 = 1
**Items included:**
Power outages<br>Fossil fuel prices<br>Electricity prices<br>Electricity use per capita<br>Imported electricity<br>Societal conflicts over energy infrastructure<br>Energy related international controversies

4. Complete ANOVA tables

a. Socio-demographics

Table S4. Differences between the energy system expectation clusters with respect to socio-demographics, future orientation and political orientation.

	Cluster 1		Cluster 2		Cluster 3		Cluster 4		ANOVA	
	M	*SD*	*M*	*SD*	*M*	*SD*	*M*	*SD*	*F*	*p*
Socio-demographics										
Women (N=639)	.55	*.50*	.47	*.50*	.47	*.50*	.49	*.50*	.79	.499[1]
Age (years)	45.33	15.1	46.72	15.0	43.65	14.5	43.13	15.4	2.12	.097
Children (average)	1.96	1.12	2.06	1.31	1.94	1.17	1.93	1.25	.39	.27
Education (7pt, higher equals more formal education)	4.99	1.37	5.02	1.51	4.82	1.43	5.09	1.40	.86	.460
Household income	4.53	3.36	4.91	3.53	4.49	3.51	4.58	3.37	.61	.612
Full time job (N=272)	.42	.50	.39	.49	.41	.49	.48	.50	.99	.369
Part time job (N=109)	.20	.41	.16	.36	.16	.36	.17	.38	.55	.652
Self-employed (N=47)	.07	.26	.08	.27	.07	.25	.07	.26	.08	.970
Unemployed (N=36)	.04	.19	.06	.23	.12	.33	.03	.16	4.75	.003
Retired (N=98)	.14	.35	.19	.39	.11	.31	.16	.37	1.23	.274
Student (N=)	.09	.29	.07	.26	.07	.26	.08	.27	.25	.862
Unable to work (N=)	.03	.17	.04	.20	.04	.20	.02	.13	.73	.535
Home owner (N=)	1.72	.50	1.71	.51	1.71	.51	1.59	.56	2.28	.078
Access to car in household	1.32	.48	1.18	.42	1.24	.43	1.16	.38	4.38	.005
Future and political orientation										
CFC 12-point (higher equals more future orientation)	58.6	7.44	55.7	7.80	52.2	7.00	55.0	7.87	15.8	.000

Left/right leaning on the political scale (5 point scale)	2.86	.99	3.20	.94	3.18	.92	3.05	.99	3.84	.010
Self-assessed familiarity with CH politics	5.73	1.57	5.64	1.72	4.81	1.79	5.36	1.93	7.30	.000
Self-assessed political activity	4.49	1.79	4.53	1.70	4.07	1.80	4.28	1.92	1.99	.114
Belief in value of voting (My vote makes a difference)	4.36	1.78	4.04	1.63	3.72	1.64	4.28	1.73	3.86	.009
Trust in parliament	4.27	1.51	3.88	1.44	3.69	1.46	4.34	1.36	7.07	0.000
Trust in energy minister	4.10	1.71	3.62	1.70	3.62	1.49	4.20	1.65	5.73	.001
Trust in science	5.32	1.19	4.89	1.28	4.18	1.43	5.05	1.23	18.71	.000

Notes. *M*=Mean, *SD*=Standard Deviation. *F*= variance of the group means, *p*=significance.

b. Energy attitudes

Table S5: Differences between the energy system expectation clusters with respect to energy attitudes and voting behaviour in the ES2050 referendum.

Attitudes towards energy	*Cluster 1*		*Cluster 2*		*Cluster 3*		*Cluster 4*		*ANOVA*	
	M	*SD*	*M*	*SD*	*M*	*SD*	*M*	*SD*	*F*	*p*
Perceived need of an energy transition	5.65	1.46	5.09	1.48	4.53	1.46	5.14	1.40	12.89	.000
Preference for locally produced electricity	4.80	1.64	4.74	1.53	4.09	1.54	4.69	1.45	6.01	.000
Willingness to sacrifice landscape aesthetics in order to place energy infrastructure	4.81	1.61	4.49	1.62	4.24	1.53	4.78	1.42	4.24	.006
My local surroundings are already impacted by energy infrastructure	2.93	1.42	3.24	1.57	3.21	1.34	3.19	1.53	1.32	.269
Energy infrastructure impacts me more than others	4.78	1.33	4.79	1.33	4.30	1.37	4.76	1.38	4.04	.007
Energy topics fascinate me	4.86	1.44	4.87	1.31	4.53	1.38	4.80	1.45	1.69	.167
Energy topics annoy me	3.98	1.53	4.46	1.48	4.25	1.41	4.04	1.45	3.85	.009
Support for photovoltaics	6.49	1.01	6.01	1.06	5.12	1.57	6.10	1.10	30.68	.000

Support for hydropower	5.88	1.46	5.73	1.11	5.25	1.32	5.62	1.28	5.61	.001
Support for wind	5.99	1.26	5.31	1.55	4.74	1.75	5.39	1.53	14.71	.000
Support for deep geothermal energy	4.22	1.87	4.01	1.85	3.80	1.53	4.47	1.60	4.20	.006
Support for gas	3.28	1.58	3.28	1.58	3.48	1.54	3.45	1.50	.42	.742
Support for nuclear	1.94	1.35	2.79	1.86	2.90	1.64	2.62	1.59	9.54	.000
Support for Electricity imports	2.79	1.29	2.91	1.31	3.43	1.46	2.93	1.38	5.76	.001
ES2050 yes (N=191)	.41	.49	.26	.44	.20	.41	.33	.47	5.29	.001
ES2050 no (N=100)	.08	.27	.22	.41	.16	.37	.14	.35	3.88	.009
ES2050 did not vote (N=125)	.15	.36	.21	.41	.25	.44	.17	.38	1.72	.161
ES2050 not allowed to vote (N=55)	.12	.32	.07	.25	.07	.26	.09	.29	1.05	.370
Es2050 cannot remember (N=112)	.17	.38	.18	.38	.17	.38	.18	.39	.040	.989
ES2050 do not want to disclose (N=55)	.07	.26	.07	.26	.12	.33	.08	.27	1.08	.354

Notes. *M*=Mean, *SD*=Standard Deviation. *F*= variance of the group means, *p*=significance.

5. Questionnaire with translated survey items

The following tables list survey items used in our study. Please note that the survey was fielded in German. English translations are provided below the German version. The left column refers to the label of the respective question in the .csv file available at: 10.5281/zenodo.3395054

a. Quota filter page

Willkommen zur Umfrage! Vor dem Start benötigen wir einige Angaben über Sie.

Welcome to the survey! Before we start we need some information about you.

Table S6 Questionnnaire with survey items (german and English Translation).

v_3	Geschlecht<br><br>Gender	männlich<br>weiblich<br>keine Angabe<br>male<br>female<br>no answer	1<br>2<br>3
v_4	Geburtsjahr<br><br>Year of birth	--- Bitte wählen Sie ---<br>1936 oder früher<br>1937<br>…<br>2000<br>2001<br>2002 oder später	0<br>1<br>2<br>...<br>66<br>67

b. Welcome and informed consent

P1	**Willkommen zu dieser Umfrage der Universität Basel und der Zürcher Hochschule für Angewandte Wissenschaften (ZHAW)**<br><br>Die Befragung dauert ungefähr 15 Minuten und ist in zwei Teile gegliedert:<br><br>• In Teil 1 geht es um Ihre persönliche Meinung und Ihren Wissensstand zu verschiedenen Energiethemen.<br>• In Teil 2 geht es um allgemeine Fragen zu Ihrer Person.<br><br>Dabei ist Ihre Anonymität vollumfänglich gewährleistet. Ihre Angaben werden ausschliesslich für wissenschaftliche Zwecke verwendet.<br><br>Falls Sie Fragen oder Anregungen haben, können Sie sich gerne an Prof. Dr. Aya Kachi wenden (aya.kachi@unibas.ch)

	Herzlichen Dank für Ihre Teilnahme!<br>Welcome to this survey conducted by the University of Basel and the Zurich University of Applied Sciences (ZHAW).<br>The interview lasts about 15 minutes and is divided into two parts:<br><br>- Part 1 deals with your personal opinion and your level of knowledge on various energy topics.<br>- Part 2 is about general questions about your person.<br>Your anonymity is fully guaranteed. Your details will be used exclusively for scientific purposes.<br>If you have any questions or suggestions, please feel free to contact Prof. Dr. Aya Kachi (aya.kachi@unibas.ch).<br>Thank you very much for your participation!

[Page break]

c. Energy Expectations [2050 for Main and 2030 for Experimental sample]

Wenn Sie die politischen und wirtschaftlichen Realitäten berücksichtigen, wie stellen Sie sich die Energiezukunft im Jahr 2050[main sample]/2030 vor?

If you consider the political and economic realities, how do you envisage the energy future in 2050 [main sample]/2030[experimental sample]?

Var. Name	Var. Label	ITEM	SCALE	NUMERICS
V_709	A_ES2030_erneuerbare	Der Anteil erneuerbarer Energien an der Stromproduktion ist im Vergleich zu heute...<br>The share of renewable energies in electricity production is in comparison to today's market share....	Deutlich geringer<br>Significantly lower<br>…<br>…<br>Genau gleich<br>exactly the same<br>…<br>…<br>Deutlich höher<br>Significantly higher	1<br>2<br>3<br>4<br>5<br>6<br>7
V_710	A_ES2030_effizienz	Die Effizienz technischer Geräte, Maschinen und Prozesse ist im Vergleich zu heute...<br>The energy efficiency of appliances and processes is in comparison to today's technology...		
V_711	A_ES2030_FZ	Der Anteil elektrischer Fahrzeuge ist im Vergleich zu heute...<br>The proportion of electric vehicles is in comparison to today....		
V_712	A_ES2030_ProKopf	Der Stromverbrauch pro Kopf ist im Vergleich zu heute...		

		The power consumption per capita is in comparison to today....		
V_713	A_ES2030_Strompreis	Der Preis für Strom ist im Vergleich zu heute...<br>The price of electricity is in comparison to today...		
V_714	A_ES2030_gas_oel_preis	Der Preis für Öl und Gas ist im Vergleich zu heute...<br>The price for oil and gas is in comparison to today...		
V_715	A_ES2030_MengeImportstrom	Die Menge an importiertem Strom ist im Vergleich zu heute...<br>The amount of imported electricity is in comparison to today...		
V_716	A_ES2030_Stromausfaelle	Die Häufigkeit von Stromausfällen ist im Vergleich zu heute...<br>The frequency of power outages is in comparison to today...		
V_717	A_ES2030_konfl_nach2160barn	Die Häufigkeit von energiepolitischen Interessenskonflikten mit Nachbarländern ist im Vergleich zu heute...<br>The frequency of controversies with neighbouring countries is in comparison to today...		
V_718	A_ES2030_konfl_gesellschaft	Die Häufigkeit gesellschaftlicher Konflikte beim Ausbau der Schweizer Energieinfrastruktur (z.B. Kraftwerke oder Stromleitungen) ist im Vergleich zu heute...<br>The frequency of societal conflicts in Switzerland over energy infrastructure (e. g. power plants or power lines) is in comparison to today...		

d. Experimental sample: Idealistic and realistic framing

1) Realistic:

Wenn Sie wiederum die politischen und wirtschaftlichen Realitäten berücksichtigen, wie hoch denken Sie wird der Anteil erneuerbarer Energien am Gesamtenergieverbrauch der Schweiz (inklusive Wärme und Mobilität) im Jahr 2050 sein?

When you again consider the political and economic realities, how high do you think the share of renewable energies in Switzerland’s total energy consumption (including heat and mobility) will be in 2050?

2) Idealistic

Wie hoch sollte Ihrer Meinung nach der Anteil erneuerbarer Energien am Gesamtenergieverbrauch der Schweiz (inklusive Wärme und Mobilität) im Jahr 2050 sein?

In your opinion, how high should the share of renewable energies in Switzerland's total energy consumption (including heat and mobility) be in 2050?

Var. Name	Var. Label	ITEM	SCALE	NUMERICS

v_720	A_Renewable_Anteil_Realistisch	*Bewegen Sie den Schieberegler an die gewünschte Position, der entsprechende Prozentanteil wird dann angezeigt. Zum Vergleich: Gemäss Bundesamt für Energie (BFE) betrug der erneuerbare Anteil im Jahr 2016 rund 21%.*<br>Move the slider to the desired position and the corresponding percentage will be displayed. By way of comparison, according to the Swiss Federal Office of Energy (SFOE), the share of renewable energy was around 21% in 2016.	1<br>…<br>100	1<br>…<br>100
V_127	B_Renewable_Anteil_Idealistisch	*Bewegen Sie den Schieberegler an die gewünschte Position, der entsprechende Prozentanteil wird dann angezeigt. Zum Vergleich: Gemäss Bundesamt für Energie (BFE) betrug der erneuerbare Anteil im Jahr 2016 rund 21%.*<br>Move the slider to the desired position and the corresponding percentage will be displayed. By way of comparison, according to the Swiss Federal Office of Energy (SFOE), the share of renewable energy was around 21% in 2016.	1<br>…<br>100	1<br>…<br>100

e. Energy attitudes

Var. Name	Var. Label	ITEM	SCALE	NUMERICS
v_30	BC_lokalStrom	Lokal produzierter Strom ist mir lieber, als Strom der nicht aus der Region kommt.<br>I prefer locally produced electricity.	1 stimme überhaupt nicht zu<br>Completely disagree<br>Stimme nicht zu<br>Disagree<br>Stimme eher nicht zu<br>Rather disagree<br>Teils/Teils<br>Partly/Partly<br>Stimme eher zu<br>Rather agree<br>Stimme zu<br>Agree<br>7 Stimme voll und ganz zu<br>Completely agree	1<br>…<br>7

v_32	BC_Landschaftsbild	Ich bin bereit, Veränderungen des Schweizer Landschaftsbildes in Kauf zu nehmen, um die Stromversorgung sicherzustellen.<br><br>I am willing to accept changes in the Swiss landscape in order to secure electricity supply.		
v_33	BC_Energiewende	Wir brauchen eine Energiewende.<br><br>We need an energy transition.		
v_35	BC_lokaleumgebungbeeinraechtigt	Meine lokale Umgebung ist stark von Energieinfrastruktur (z.B. Kraftwerke oder Stromleitungen) beeinträchtigt.<br><br>My local environment is severely affected by energy infrastructure (e. g. power plants or power lines).		
v_36	BC_energieinfraAndereMehr	Energieinfrastruktur (z.B. Kraftwerke oder Stromleitungen) beeinträchtigt andere Leute mehr als mich.<br><br>Energy infrastructure impacts others more than me		
v_142	BC_spannend	Ich finde Energiethemen spannend.<br><br>Energy topics fascinate me.		
v_143	BC_aergern	Ich ärgere ich mich oft über Energiethemen.<br><br>Energy topics annoy me.		

Inwiefern stimmen Sie den folgenden Aussagen zu?
Um die Schweizer Stromversorgung in der Zukunft zu gewährleisten, sollen neue Anlagen des folgenden Energietyps gebaut werden.

To what extent do you agree with the following statements?
In order to guarantee Switzerland's future electricity supply, new plants of the following energy type are to be built.

**Var. Name**	**Var. Label**	**ITEM**	**SCALE**	**NUMERICS**
v_20	BC_zustimmung_solarstrom	Solarstrom<br><br>Solar	1 stimme überhaupt nicht zu<br><br>Completely disagree<br><br>Stimme nicht zu<br><br>Disagree<br><br>Stimme eher nicht zu<br><br>Rather disagree<br><br>Teils/Teils	1<br><br>…<br><br>7

			Partly/Partly<br>Stimme eher zu<br>Rather agree<br>Stimme zu<br>Agree<br>7 Stimme voll und ganz zu<br>Completely agree	
v_21	BC_zustimmung_HP	Wasserkraft<br>Hydropower		
v_22	BC_zustimmung_wind	Windenergie<br>Wind power		
v_24	BC_zustimmung_DGE	Tiefengeothermie<br>Deep geothermal		
v_25	BC_zustimmung_Gas	Gas<br>Gas		
v_37	BC_zustimmung_nuklearenergie	Nuklearenergie<br>Nuclear energy		
v_450	BC_zustimmung_importVsAusbauProduktionsanalgen	Strom importieren ist sinnvoller als ein weiterer Ausbau der Schweizer Energieproduktionsanlagen.<br>Importing electricity makes more sense than further expanding Swiss energy production facilities.		

f. Political orientation and trust

Var. Name	Var. Label	ITEM	SCALE	NUMERICS
v_299	ABC_CHPolitk	Ich setze mich oft mit Schweizer Politik auseinander.<br>I generally follow Swiss politics. (Self-assessed familiarity with CH politics)	1 stimme überhaupt nicht zu<br>Completely disagree<br>Stimme nicht zu<br>Disagree<br>Stimme eher nicht zu<br>Rather disagree	1<br>…<br>7

			Teils/Teils<br>Partly/Partly<br>Stimme eher zu<br>Rather agree<br>Stimme zu<br>Agree<br>7 Stimme voll und ganz zu<br>Completely agree	
v_300	ABC_PolAktiv	Ich halte mich für politisch aktiv.<br>I am politically active (Self-assessed political activity)		
v_737	ABC_links_rechts	Wie stufen Sie Ihre politische Haltung auf einer Skala ein?<br>How do you rate your political stance on a scale?	1 (links)<br>1 (left)<br>…<br>5 (right)<br>5 (rechts)	1<br>…<br>5
v_8	ABC_SVP	Welche Partei vertritt am ehesten Ihre politische Meinung?<br>Which party most closely represents your political opinion?	Schweizerische Volkspartei (SVP)	0 = not quoted<br>1=quoted
v_10	ABC_SP		Sozialdemokratische Partei der Schweiz (SP)	
v_462	ABC_GPS		Grüne Partei (GPS)	
v_11	ABC_GLP		Grünliberale Partei (GLP)	
v_12	ABC_FDP		FDP.Die Liberalen (FDP)	
v_13	ABC_EVP		Evangelische Volkspartei (EVP)	
v_14	ABC_CVP		Christlichdemokratische Volkspartei (CVP)	
v_15	ABC_AL		Alternative Liste (AL)	
v_16	ABC_EDU		Eidgenössisch-Demokratische Union (EDU)	
v_17	ABC_Poluninteressiert		Ich bin politisch uninteressiert<br>I am politically uninterested	

v_18	ABC_Keineantwort		Darauf möchte ich nicht antworten<br>Do not want to answer	
v_19	ABC_AnderePartei		Andere Partei<br>Other party	
v_407	ABC_ES2050_JA	Wie haben Sie im Mai 2017 bei der Abstimmung zur Energiestrategie 2050 abgestimmt?<br>How did you vote on the ES2050 in May 2017?	Ich habe für die Annahme der Energiestrategie 2050 gestimmt.<br>I voted for the acceptance of the ES2050.	
v_408	ABC_ES2050_Nein		Ich habe für die Ablehnung der Energiestrategie 2050 gestimmt.<br>I voted to dismiss the ES2050.	
v_409	ABC_ES2050_NoVote		Ich habe nicht abgestimmt.<br>I did not vote.	
v_410	ABC_ES2050_Noallowed		Ich bin nicht stimmberechtigt.<br>I am not entitled to vote.	
v_416	ABC_ES2050_Keinantwort		Darauf möchte ich nicht antworten.<br>I do not want to answer.	
v_433	ABC_ES2050_Keinerinnerung		Ich kann mich nicht erinnern.<br>I cannot remember.	
v_305	BC_Vertrauen_Einfluss	Meine Stimme hat einen Einfluss auf die Schweizer Politik.<br>My vote makes a difference (Belief in value of voting)	1 Stimme nicht zu<br>Disagree<br>Stimme eher nicht zu<br>Rather disagree<br>Teils/Teils<br>Partly/Partly<br>Stimme eher zu<br>Rather agree<br>Stimme zu<br>Agree<br>7 Stimme voll und ganz zu<br>Completely agree	1<br>…<br>7

v_307	BC_Vertrauen_parlament	Ich habe Vertrauen in das Schweizer Parlament.<br>I trust the Swiss parliament.		
v_308	BC_Vertrauen_Doris	Ich habe Vertrauen in Doris Leuthard (Vorsteherin des Departements Umwelt, Verkehr, Energie und Kommunikation (UVEK).<br>I trust Doris Leuthard (Swiss Energy minister)		
v_309	BC_Vertrauen_Wissenschaft	Ich habe Vertrauen in die Wissenschaft und wissenschaftliche Forschungsergebnisse.<br>I trust science and its research results.		

g. CFC Scale

Bei den folgenden Fragen geht es um Ihre persönliche Einstellung gegenüber zukünftigen Ereignissen.

Entscheiden Sie, wie stark Sie den Aussagen zustimmen. Es gibt keine richtigen oder falschen Antworten.

The following questions are about your personal attitude towards future events.

Decide how strongly you agree with the statements. There are no right or wrong answers.

Var. Name	Var. Label	ITEM	SCALE	NUMERICS
v_51	BC_CFC_1	Ich überlege mir oft, wie die Zukunft aussehen könnte.<br>I consider how things might be in the future, and try to influence those things with my day to day behavior.	1 Stimme nicht zu<br>Disagree<br>Stimme eher nicht zu<br>Rather disagree<br>Teils/Teils<br>Partly/Partly<br>Stimme eher zu<br>Rather agree<br>Stimme zu<br>Agree<br>7 Stimme voll und ganz zu<br>Completely agree	1<br>…<br>7
v_52	BC_CFC_2	Bequemlichkeit spielt in meinen Entscheidungen eine wichtige Rolle.<br>My convenience is a big factor in the decisions I make or the actions I take.		
v_53	BC_CFC_3	Ich bin bereit zu verzichten, wenn es dem Erreichen zukünftiger Ziele dient.		

		I am willing to sacrifice my immediate happiness or well-being in order to achieve future outcomes.		
v_54	BC_CFC_ 4	Normalerweise ist es nicht nötig, auf etwas zu verzichten, da man sich um zukünftige Folgen auch noch später kümmern kann.<br><br>I think that sacrificing now is usually unnecessary since future outcomes can be dealt with at a later time. (I)		
v_55	BC_CFC_ 5	Ich halte es für wichtig, Warnungen vor negativen Folgen ernst zu nehmen, auch wenn die Folgen erst in vielen Jahren auftreten werden.<br><br>I think it is important to take warnings about negative outcomes seriously even if the negative outcome will not occur for many years.		
v_56	BC_CFC_ 6	Oft ignoriere ich Warnungen vor möglichen zukünftigen Problemen, weil diese oft gelöst werden, bevor es zur Krise kommt.<br><br>I generally ignore warnings about possible future problems because I think the problems will be resolved before they reach crisis level.		
v_57	BC_CFC_ 7	Eine Handlung mit grossen langfristigen Konsequenzen ist mir wichtiger, als eine Handlung mit kleinen aber unmittelbaren Konsequenzen.<br><br>I think it is more important to perform a behavior with important distant consequences than a behavior with less important immediate consequences.		
v_58	BC_CFC_ 8	Da meine alltäglichen Handlungen konkrete Auswirkungen haben, sind sie mir wichtiger, als Handlungen die erst in ferner Zukunft Auswirkungen haben.<br><br>Since my day to day work has specific outcomes, it is more important to me than behavior that has distant outcomes		
v_739	BC_CFC_ 9	Ich bemühe mich oft Ergebnisse zu erreichen, die erst in vielen Jahren eintreten.		

		Often I engage in a particular behavior in order to achieve outcomes that may not result for many years.		
v_740	BC_CFC_ 10	Ich handle nur, um meine jetzigen Bedürfnisse zu befriedigen. Die Zukunft wird die Dinge schon selbst regeln.<br>I only act to satisfy immediate concerns, figuring the future will take care of itself.		
v_741	BC_CFC_ 11	Mein Verhalten wird nur von den absehbaren Ergebnissen meiner Handlungen beeinflusst (im Hinblick auf Tage oder Wochen).<br>My behavior is only influenced by the immediate (i.e., a matter of days or weeks) outcomes of my actions.		
v_742	BC_CFC_ 12	Ich handle nur um meine jetzigen Bedürfnisse zu befriedigen, um zukünftige Probleme kann ich mich auch später noch kümmern.<br>I only act to satisfy immediate concerns, figuring that I will take care of future problems that may occur at a later date		

h. Demographics

v_5	ABC_Eigentuemer_y_n	Sind Sie im Besitz der Liegenschaft bzw. der Wohnung, in der Sie leben?<br>Are you owning the property or apartment in which you live?	1 Yes<br>2 No<br>3 Do not want to answer	
v_7	ABC_auto	Besitzen Sie oder eine Person in Ihrem Haushalt ein Auto?<br>Do you or a person in your household own a car?	1Ja<br>1 Yes<br>2 Nein<br>2 No<br>3 Ich möchte nicht antworten<br>3 Do not want to answer	
v_310	ABC_Kinder	Haben Sie Kinder?<br>Do you have kids?	1 Keine None<br>2 1<br>3 2<br>4 3<br>5 4<br>6 5 or more	

			7 Do not want to answer.	
v_126	ABC_Bildung	Welches ist Ihr höchster Bildungsabschluss?<br>What is your highest level of education?	1 kein Schulabschluss<br>2 Obligatorische Schule<br>3 Anlehre, Haushaltsjahr<br>4 Berufslehre, Berufsschule, Vollzeitberufsschule, Handelsschule<br>5 Berufsmatura, Maturitätsschule, Lehrerseminar, Diplom- und Wirtschaftsmittelschule<br>6 Meisterprüfung, Techniker- und Fachschule, höhere Fachschule, Ingenieurschule, Technikum<br>7 Fachhochschule, Universität, ETH, Doktorat<br>8 Andere Ausbildung<br>9 Darauf möchte ich nicht antworten.	
v_463	ABC_Vollzeit	Wie sieht ihre berufliche Situation derzeit aus?<br>What is your current professional situation?	Vollzeitangestellt<br>Full-time employed.	
v_464	ABC_Teilzeit		Teilzeitangestellt<br>Part-time employed.	
v_465	ABC_Selbststaendig		Selbstständig<br>Self-employed	
v_466	ABC_Arbeitslos		Arbeitslos<br>Unemployed.	
v_467	ABC_Pensioniert		Pensioniert<br>Retired	
v_468	ABC_Student		Student/-in<br>Student	
v_469	ABC_Arbeitsunfaehig		Arbeitsunfähig<br>Unable to work	
v_470	dupl1_ABC_KeineAntwort		Darauf möchte ich nicht antworten.<br>I do not want to answer.	
v_313	ABC_einkommen	Wie hoch ist das monatliche Nettoeinkommen Ihres Haushalts? (Einkommen aller Haushaltsmitglieder nach Abzügen)	1 Weniger als 4'000 Fr.<br>2 4'001-6'000 Fr.<br>3 6'001-8'000 Fr.	

		What is the monthly net income of your household? (income of all household members after deductions)	4 8'001-10'000 Fr.<br>5 10'001-12'000 Fr.<br>6 12'001-14'000 Fr.<br>7 14'001-16'000 Fr<br>8 16'001-18000 Fr.<br>9 Mehr als 18'000 Fr.<br>10 Darauf möchte ich nicht antworten.	
