# Supplementary material for: How the public imagines the energy future: Exploring and clustering non-experts’ techno-economic expectations towards the future energy system
Source: PLoS One. 2020 Mar 5;15(3):e0227369. doi: 10.1371/journal.pone.0227369 (PMC7058329; doi:10.1371/journal.pone.0227369)
Supplement: Attachment — Submitted filename: Response to reviewers.docx [file pone.0227369.s002.docx]

# Dear Editor,

# Dear Reviewers.

# We like to thank the two reviewers as well as the editor for their time and effort to provide us with a very helpful feedback. Apart from pointing out specific mistakes and passages that require clarification, they also motivated us to improve some aspects of the manuscript more systematically.

# In particular, we addressed two main points raised by both reviewers. First, we extended our description of the sampling process and provide more information on how the sample compares to the general population in Switzerland. Second, we revisited the manuscript to achieve a better alignment of discussion and data.

# Our answer to each review comment and corresponding actions can be found below. In these answers, all changes that concern the content of our manuscript are reflected. Typos and small mistakes that we spotted during the general overhaul of our submission are not listed individually, but are documented in the track-changes version of the manuscript. The same applies to changes that are associated with an adaption of the manuscript to the formal guidelines of PLOS One, which mainly concerned headings and the title page.

# We feel that the revision process has improved our paper’s quality significantly and are confident that it is now on a level that makes it a relevant and adequate research contribution. These improvements would not have been possible without the valuable and constructive feedback we received. We are extremely grateful for this.

# Sincerely on behalf of all authors,

# Lukas Braunreiter

| **Nr./**  **Reviewer** | **Comment** | **Response and action** |
| --- | --- | --- |
| #1  Editor | Please ensure that your manuscript meets PLOS ONE's style requirements, including those for file naming. | We have adapted author affiliations to meet PLOS One formatting guidelines.  In particular, we adapted the title to make it more specific about the study’s scope and methodology.  We also adapted heading formatting throughout the manuscript to meet PLOS One formatting guidelines. |
| #2  Editor | We noted in your submission details that a portion of your manuscript may have been presented or published elsewhere. Please clarify whether this [conference proceeding or publication] was peer-reviewed and formally published. If this work was previously peer-reviewed and published, in the cover letter please provide the reason that this work does not constitute dual publication and should be included in the current manuscript. | Empirical basis for this manuscript is a longer survey with different parts, one of which was also used for an already published peer-reviewed publication^[1]^ in *Energy Research and Social Science* (ERSS). This manuscript is using a different part of the survey, which was not covered by the published paper. We clearly state this in the manuscript. In the ERSS publication, the acceptability of hydropower and deep geothermal energy infrastructure was analysed. The energy system expectation items, which are the key focus of this manuscript, are not explored in detail in the ERSS study. This double use of surveys but still keeping uniqueness of subdata sets for distinct publications is common practice in social science to reduce response burden for respondents and survey costs for researchers.  All parts of this manuscript, including the data analysis, provide original content and insights that were not published anywhere else. As requested, we also added this explanation to the cover letter. |
| #3  Reviewer 1 | I really enjoyed this article and as far as I believe the survey and follow up analyses were sound. I would love to know why you only chose a sample from the Swiss-German speaking community, and how did you select and engage this sample. Also, although not necessary for this paper, it would have been highly insightful to have had a comparative sample from another area of Switzerland. | Thank you!  Survey participants were recruited via an online panel. Participation in the survey was voluntary, participants were assured that data was going to be analysed and published only in an anonymous way and for scientific purposes. Panel members received an invitation to participate in the study, with a small incentive of 0.75 Euro credited upon completion of the survey. This information has been added to the Method section. The sample corresponds to the general Swiss population in terms of age and gender (see comment #9).  Given the exploratory nature of our study, we decided to focus on the German speaking population. However, we completely agree that inter-language or regional comparisons as well as comparisons with other countries, where the discourse around the energy transition might be shaped by other issues, would be highly insightful. We also specifically mention this as future research option in the critical reflection of our manuscript.  Furthermore, we only include survey participants from the German speaking part of Switzerland due to practical and funding reasons. In particular, including the French and Italian speaking parts of Switzerland would have resulted in significantly higher costs. The reason for this is that the French and Italian speaking population of Switzerland is a lot smaller compared to the German speaking community. Accordingly, achieving a representative sample from these language groups would have been rather expensive. In addition, translation costs are substantially, given that forward-backward translation would have been necessary to secure identical contents.  Also, we adapted and expanded our description of the data acquisition and sampling process. For more details, see comment #9, where we also expand on the exploratory nature of this study. . |
| #4  Reviewer 1 | Sometimes the conclusions seem speculative rather than solidly derived from the data - however I understand the nature of survey analysis and that this often is the case. Also I feel that the conclusion should be revised and made a little more obvious about the outcomes/conclusions of the survey. | It is true than we cannot infer causation from our results. This is particularly the case for the comparison of the energy system expectations with the energy scenario projections. This is why we omitted two rather speculative arguments about their interdependency from section 4.3:   1) ~~Accordingly, there co-exists a range of expectations about the energy system that are more or less compatible with, and maybe more or less influenced by, the scenario constituting the national energy strategy.~~ 2) ~~In addition, many scenario characteristics are counterintuitive. While this study included the effect of long time horizons, future research could focus on the comprehensibility of the “what-if” methodology, the interdependency of models and assumptions, or the resulting plurality of possible futures. It is thus doubtful whether the publication of energy scenarios benefits public energy policy debates. Scenario-based results are typically contingent on the context (e.g., assumptions, model structure, etc.) that experts need to be aware of when applying scenarios (Fortes, Alvarenga, Seixas, & Rodrigues, 2015). The energy system expectations assessed in this study, in contrast, suggest that the public attributes meaning to particular promises and concerns detached from the scenario development context. Instead, broader world views and attitudes beyond energy preferences seem to matter.~~   In addition, we omitted two potential explanations for the differences between the 2030 and 2050 expectations in section 4.1 (please see comment #8 for details).  We agree the conclusions did not convey the main results of the survey as accurately as they could have. Accordingly, we have significantly adapted this section. We now stay much closer to the data, do not refer to methodological controversies associated with energy scenarios that were not the focus of our research and tried to improve the argumentation why the assessment of public energy system expectations is worthwhile from our perspective, i.e. because…   - …the assessment of expectations has so far predominantly been conducted among expert communities - …it has been shown that expectations can influence the acceptability of energy infrastructure and policies - …expectations and scenarios are interrelated but often understudied phenomena, that are only recently being explored, mainly in Science and Technology studies (STS) focusing on particular technology developments - An energy transition is inherently future-oriented, which is why it makes sense to compare it to future-oriented beliefs among the public |
| #5  Reviewer 1 | Also, throughout the article and particularly within the conclusion there are some English language errors - easily corrected - but worth revising especially to enhance the meaning of the conclusion. | Thank you for pointing this out. Although these are seemingly minor errors, they can have a great effect in confusing or distracting readers. We hope to have spotted all typos and spelling errors in the revised version of the manuscript (see also comment #10 from Reviewer 2). |
| #6  Reviewer 1 | As far as I understand the survey is available but the actual survey returns are not on the system. I am not sure if this is standard (?). | Yes, the survey is available in the supplementary material and it includes English translations from the original German survey items as well.  The raw data set with the survey returns is now also available at the Zenodo repository: https://zenodo.org/record/3395054#.XdA26dWDPb0 |
| #7  Reviewer 2 | This paper describes an interesting exploratory study on the role of the non-expert public in energy futures and energy transitions, with Switzerland serving as a case study. The use of cluster analysis seemed useful and appropriate. | Thank you! |
| #8  Reviewer 2 | The study findings that there were few differences between the energy systems expectations for 2030 vs. 2050 was especially interesting, though I would caution the authors in general, and in this instance in particular, to not speculate too much on the meaning behind findings such as these that they cannot explain by their analysis. | As the occasional tendency towards speculation was already pointed out by reviewer one, we tried to thoroughly address this critique in the revision process. On the one hand, we adapted two paragraphs in section 4.3 and we completely restructured the conclusions, which should now better reflect the main results of the study (please see comment # for details).  Specific to the comparison of the 2030 and 2050 energy system expectations, we omitted two reasons why the latter is more positively associated from section 4.1 because our data did not provide insights directly related to these explanations. The following paragraphs were removed: *~~One potential explanation is that the public is aware that an energy transition is associated with challenges that need to be resolved to achieve a sustainable energy future (Butler, Demski, Parkhill, Pidgeon, & Spence, 2015; Chilvers & Longhurst, 2016). Another possibility is that the public simply has more positive views of the distant future than on 2030 which is closer to the present, as it has been suggested in the literature on future orientation (Arnocky, Milfont, & Nicol, 2014; Demarque, Apostolidis, & Joule, 2013)~~.* |
| #9  Reviewer 2 | My main concern about this paper was the very limited information provided about the survey sample, respondents, and its representativeness of the general population. Reference was made to more detailed descriptions of the questionnaire and the participant recruitment process in Blumer et al. 2018, but some of that detail should be described in this paper too. For instance, why was only German speaking populations in the country recruited, and not French speaking populations in Switzerland as well? Did the authors attempt to develop a random or stratified random sample that was representative of the general population, and if not why not? How successful were they in this effort? | We agree that the information provided on the sample and its representativeness of the general population was too minimalistic in the original submission. Accordingly, we have adapted and expanded the sample description.  We applied quota sampling for the categories age and gender. In particular, five age categories (18-29; 30-39; 40-49; 50-59, 60+) were defined per gender. Once a quota was filled, additional respondents belonging to the category were screened out at the beginning of the survey. In total, 35 participants had to be screened out.  Due to the application of these quota, the sample corresponds to the general Swiss population in terms of age and gender. Additionally, it turns out that also the political party identification (especially for the four largest political parties) and the share of homeowners compare very well to the general Swiss population. The share of university degree holders is slightly lower in our sample, compared to the Swiss population (22.7% in the sample, as opposed to 27% in the Swiss population). Hence, for the main characteristics known to be influencing acceptability and preferences for energy policies and infrastructure (e.g. age, gender, political orientation), our sample generally compares very well to the Swiss population.  For the reasoning why we only were able to include German speaking survey participants, please see comment #3. As stated in the manuscript [see section 1.4], the goal of the paper at hand was not to provide a representative overview, but to explore the how expectations of the energy future can be operationalized among non-experts. Further research should indeed go beyond this and consider differences between subsamples of the population more generally. |
| #10  Reviewer 2 | The paper was generally well written but I found a few exceptions:  line 296, "increase more s than the share ..." means what? line 474, "The variance among in the expectations ..." means what? line 519, "thus considered to be transition optimists" - would it be clearer to say "thus be considered transition optimists." ? line 562, "Clusters and Projections", not "Clusters ad Projections" line 608, "I think you mean "factors that typically are outside the", not "factors that typically is outside the" | Thank you very much for spotting these mistakes and listing them here, which made it very convenient for us to correct them. Your suggestions were spot on.  We found a few more spelling errors throughout the manuscript during the revision process. All of them can be seen in the track-changes version of the manuscript. |

1. Blumer, Y. B., Braunreiter, L., Kachi, A., Lordan-Perret, R., & Oeri, F. (2018). A two-level analysis of public support: Exploring the role of beliefs in opinions about the Swiss energy strategy. *Energy Research & Social Science*, *43*, 109-118. DOI: 10.1016/j.erss.2018.05.024
